# OCT-Based Morphological Classification of Healed Coronary Plaques: Insights from Imaging of Fresh Thrombi at Different Stages of Healing and Implications for Post-Stenting Edge Dissections

**DOI:** 10.3390/medicina61081440

**Published:** 2025-08-10

**Authors:** Calin Homorodean, Horea-Laurentiu Onea, Florin-Leontin Lazar, Mihai Claudiu Ober, Mihail Spinu, Dan-Alexandru Tataru, Maria Olinic, Ioana Rada Popa Ilie, Romana Homorodean, Daniel-Corneliu Leucuta, Dan-Mircea Olinic

**Affiliations:** 1 4th Department of Internal Medicine, Medical Clinic No. 1, Iuliu Hatieganu University of Medicine and Pharmacy, 400006 Cluj-Napoca, Romania; chomorodean@yahoo.com (C.H.); lazar.leontin@yahoo.com (F.-L.L.); spinu_mihai@yahoo.com (M.S.); tataru.cardio@gmail.com (D.-A.T.); maria.olinic@yahoo.com (M.O.);; 2 2nd Department of Cardiology, County Clinical Emergency Hospital Cluj-Napoca, 400006 Cluj-Napoca, Romania; mihai_ober@yahoo.com; 3 Department of Cardiology, County Clinical Emergency Hospital Sibiu, 550245 Sibiu, Romania; 4 Department of Endocrinology, Faculty of Medicine, Iuliu Hatieganu University of Medicine and Pharmacy, 400349 Cluj-Napoca, Romania; ioana.ilie@umfcluj.ro; 5 1st Department of Neurology, County Clinical Emergency Hospital Cluj-Napoca, 400012 Cluj-Napoca, Romania; romanaque@yahoo.com; 6 Department of Medical Informatics and Biostatistics, Iuliu Hațieganu University of Medicine and Pharmacy, 400349 Cluj-Napoca, Romania; dleucuta@umfcluj.ro

**Keywords:** atherosclerotic coronary disease, acute coronary syndromes, optical coherence tomography, healed coronary plaques, culprit lesions, edge dissections

## Abstract

*Background and Objectives*: In vivo data on healed coronary plaques (HCPs), the hallmark of previous plaque disruption, remains scarce. The study aimed to use optical coherence tomography (OCT) imaging to assess the prevalence, morphological features, and clinical significance of culprit HCPs in patients with acute coronary syndrome (ACS). *Materials and Methods*: A total of 87 ACS patients (74.3% non-ST-segment elevation ACS) who underwent pre-procedural OCT imaging of the culprit vessel at a single center were retrospectively analyzed. A pilot subgroup of patients with intracoronary thrombi at the culprit site, in various stages of organization and healing, enabled a detailed morphological characterization of HCP despite the absence of histological validation. Three distinct HCP imaging aspects were identified: type I—overlaying fibrous tissue, type II—overlaying lipid tissue, and type III—overlaying calcific tissue. HCP presence was subsequently assessed in the entire population. Clinical correlations included associations with post-stenting outcomes, particularly edge dissections (ED). *Results*: Culprit HCPs were identified in 78 patients (89.7%): type I—30.8%, type II—51.3%, and type III—17.9%. Regarding the underlying substrate and complication mechanism, type I HCP was associated with pathological intimal thickening (70.8%) and plaque erosion (75%), type II with lipid-rich plaque (80%) and plaque rupture (PR) (82.5%), and type III correlated with calcific plaque (92.9%, *p* < 0.0001) and both PR and calcified nodule (*p* < 0.0001). A unique signal-rich ring was observed at the HCP–tissue interface in both type II (77.5%) and type III (78.6%, *p* < 0.0001). There was a significant correlation between stent ED and HCP presence at landing zones (LZ) (HR 4.14, 95% CI: 1.79–9.55; *p* < 0.001). *Conclusions*: OCT analysis of intracoronary organizing fresh thrombi allowed detailed characterization of culprit HCPs and in vivo classification into three imaging types. This approach likely contributed to the high observed detection rate of HCP by enhancing recognition of subtle OCT features. HCP may create mechanical vulnerability if located at the stent LZ. Our improved HCP detection techniques may help optimize stent-related outcomes of OCT-guided procedures by choosing an HCP-free LZ or longer stents.

## 1. Introduction

Plaque rupture (PR), plaque erosion (PE), and, to a lesser extent, calcified nodule (CN), represent the most common mechanisms leading to occlusive vessel thrombosis, which translates clinically to the development of an acute coronary syndrome (ACS) [1]. However, when these events occur in a milieu with a favorable prothrombotic/thrombosis-resistant ratio, plaque healing can develop silently, without causing symptoms [2]. In fact, it has been demonstrated in vitro that plaque destabilization mostly occurs in this silent manner, contributing to rapid plaque progression and development of high-grade stenosis [3]. Healed coronary plaques (HCPs) exhibit pathologically a multilayered pattern composed of an organized thrombus that is gradually replaced by type III and later by type I collagen [4]. 

Optical coherence tomography (OCT) represents a high-resolution light-based intravascular imaging modality that allows ideal characterization of different tissue components and stent-related issues, superior to intravascular ultrasound in this regard, but limited by low penetration power and the need for flushing in order to remove blood [5,6]. Intravascular ultrasound continues to be supported by a larger body of evidence demonstrating its prognostic advantage in optimizing percutaneous coronary interventions [7]. Conversely, although unable to properly assess total plaque volume or deep calcium, OCT offers excellent visualization of key morphological features such as thin-cap fibroatheroma (TCFA), superficial calcium, macrophage infiltration, and coronary thrombus [5,6]. Shimokado et al. conducted a histology validation study and found that OCT is also a useful tool in detecting HCP, with a high sensitivity and specificity (81% and 98%, respectively) [8]. HCP is identified in the presence of a multilayer tissue with different optical densities [4].

Presently, in vivo data on the biological and clinical characteristics of HCP in the coronary tree is lacking. Moreover, the influence of underlying plaque morphology on HCP imaging pattern was not previously described. Recent OCT data suggests that HCPs are widespread, affecting patients with stable coronary artery disease (CAD) more frequently than ACS, although the reported incidence varies across studies (53–75% vs. 29–62%) [9,10,11]. An HCP phenotype at the ACS culprit site is associated with pan-coronary vulnerability features (including HCP presence at non-culprit sites) and more advanced atherosclerotic disease [9,12]. OCT diagnosis of HCP may be the subject of several sources of bias, particularly induced by potential misinterpretation of calcific plaques, lipid plaques, or intramural hematoma and dissection. Therefore, a detailed imaging characterization is needed.

In this study, we aim to characterize in detail the OCT morphological features of HCP with particular focus on distinguishing HCP overlaying fibrous, lipid-rich, and calcific tissues. Given the lack of histopathological evidence and potential OCT misinterpretation due to overlap with other plaque morphologies, we initiated this characterization by analyzing fresh intracoronary thrombi at various stages of organization and healing. These thrombi provide direct imaging-based evidence of HCP development. Furthermore, we seek to assess the prevalence of culprit HCP in patients with ACS and to explore their clinical significance, particularly their potential impact on stenting results, such as edge dissections (ED).

## 2. Materials and Methods

### 2.1. Study Population

Between January 2019 and January 2024, consecutive patients presenting with an ACS and an indication to perform coronary angiography followed by OCT imaging in a single tertiary center—Cluj County Emergency Hospital, Department of Interventional Cardiology were retrospectively included.

Informed written consent was obtained prior to any invasive intervention. The decision to perform OCT imaging and which vessel to explore was made entirely by the interventional cardiologist who performed the index procedure, thus representing a potential source of bias, particularly in selecting less severe ST-segment elevation myocardial infarction (STEMI) patients. The following preliminary exclusion criteria were applied: (1) poor image quality; (2) stent-related culprit lesion—in-stent restenosis/thrombosis; (3) post-interventional imaging only; (4) unidentifiable culprit lesion.

**Figure 1:**
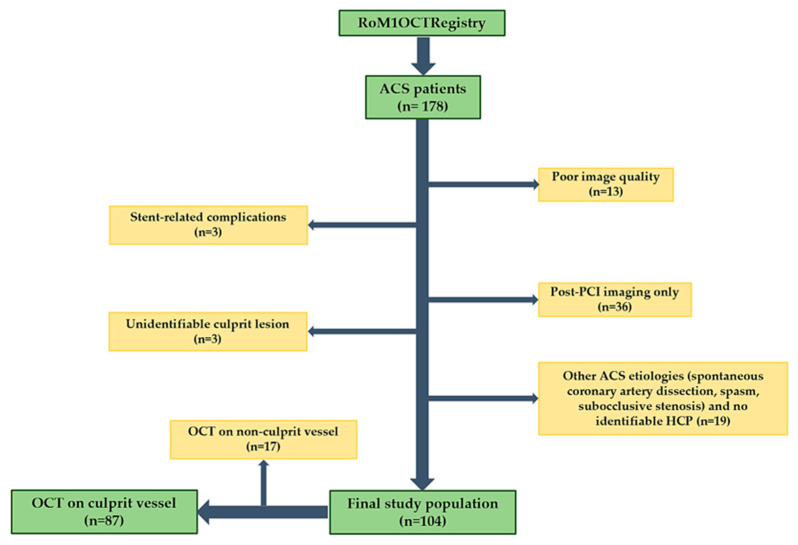
Study group.

Among the initial study population comprising 178 consecutive patients, 55 patients were excluded after applying the aforementioned criteria. Etiologies of ACS different from PR, PE, or CN, and cases where there was no identifiable HCP both at the culprit as well as non-culprit site were not included (n = 19). A total of 104 patients with analyzable OCT images of the culprit or non-culprit artery were included in the final analysis (Figure 1). Out of all these cases, OCT was performed on the culprit vessel in 87 patients, while OCT of the non-culprit vessel-only was available in 17 patients. In 6 out of these 17 patients, a large residual thrombus impaired any attempts to advance the imaging probe on the culprit vessel. OCT of both the culprit and non-culprit vessel was performed in 8 patients.

The diagnosis of ACS was made according to the 2023 European Society of Cardiology Guidelines [13] and included STEMI and non-ST-segment elevation acute coronary syndromes (NSTE-ACS). NSTE-ACS included non-ST-segment elevation myocardial infarction and unstable angina pectoris.

Clinical data, including demographics, cardiovascular risk factors, history of cardiovascular diseases, laboratory data, echocardiographic parameters, and medications, were collected from the medical charts.

The culprit lesion was first identified on baseline angiograms by three senior interventional cardiologists who performed the index coronary angiography according to current standards [5,13]. The culprit plaque was determined mainly based on electrocardiographic changes and angiographic appearance, but in cases of uncertainty, echocardiographic wall motion abnormalities were used as a supportive tool to guide localization, in conjunction with clinical judgment.

The study protocol was approved by the institutional review board (Cluj County Emergency Hospital Ethics Committee—27524, from 2022), and it complies with the Declaration of Helsinki on human research.

### 2.2. Coronary Angiogram Analysis

Coronary angiography was conducted using the available on-site angiograph: Siemens Artis Zee (Siemens Healthineers, Erlangen, Germany). The severity of coronary stenosis was evaluated both visually and through quantitative coronary analysis methods (Scientific QCA, Siemens Healthineers), with the most severe degree of stenosis being taken into account. Following coronary angiography, coronary stenosis was categorized as occlusive (100%), significant (>70%/>50% for left main), non-significant (<50%), or borderline (between 50% and 70%). Multivessel disease was defined as the presence of a significant stenosis in any of the non-culprit vessels or involvement of the left main. Coronary flow was evaluated using the Thrombolysis in Myocardial Infarction score.

### 2.3. OCT Acquisition Technique and Image Analysis

OCT imaging was performed using the frequency-domain ILUMIEN^TM^ OPTIS^TM^ OCT system and C7 Dragonfly^TM^/Dragonfly^TM^ OPTIS^TM^ monorail catheter (St. Jude Medical, St. Paul, MN, USA) as previously reported [14,15]. All OCT images were stored for offline analysis in a dedicated database, the RoM1OCTRegistry, where they were analyzed by two independent experienced interventional cardiologists (C.H. and H.L.O.) who were blinded to the angiographic and clinical data. Inter-observer discrepancies were settled by a third reviewer (D.M.O.).

Intracoronary OCT images were analyzed frame-by-frame in order to identify the culprit lesion associated with the ACS.

OCT images were analyzed using previously validated criteria for plaque characterization [4]. Overall, three plaque types were identified: fibrous, lipid, and calcific plaques. A lipid-rich plaque (LRP) is defined as a plaque containing lipid pools extending in at least two quadrants [16]. Spotty calcification (calcium arc <90°, calcium length <4 mm) could also be present [17]. TCFA identifies an LRP encapsulated by a fibrous cap measuring at its thinnest part <65 μm [4]. Within the group of fibrous plaques exhibiting high backscattering, those showing moderate light attenuation in the deeper intimal layers, close to the medial layer within less than one quadrant, were classified as pathological intimal thickening (PIT) [4]. When large calcium deposits (calcium arc >90°) are identified, the resulting entity is defined as calcific plaque [18,19]. Superficial calcified plates, defined as non-protruding sheets of calcium [15,20], were included under the calcific plaque entity.

Calcium was defined by low attenuating and low signal regions with sharply delineated borders [4].

A thrombus was defined as an irregular mass floating within the lumen or attached to the intima [5]. The thrombus was categorized as either red or white. Red thrombus was identified as an intraluminal mass with a posterior shadow, whereas white thrombus appeared as a luminal mass without posterior attenuation [16].

HCP was characterized as a low-signal/low-attenuating plaque located abluminally, with at least one layer with different optical density, a clear demarcation from the underlying tissue components, and a smooth luminal surface [8].

#### 2.3.1. Pilot Subgroup of Patients

A subgroup of patients with visible fresh thrombus at the culprit site was identified. In these cases, we subsequently searched for thrombus segments showing early signs of healing, characterized by the emergence of a stratified appearance. Patients in whom these stratified healing zones were found within areas of fresh thrombus, referred to as “HCP– thrombus continuum”, were included in a dedicated Pilot subgroup.

#### 2.3.2. Pilot Subgroup Inclusion Criteria

1. Presence of visible fresh thrombus.
  - Meeting established OCT criteria for red thrombus or white thrombus.
  - Detected in at least three consecutive OCT frames.
2. Coexistence of a stratified or layered tissue component, consistent with HCP features.
  - Located within or adjacent to the thrombus mass.
3. No clear thrombus—free interface between the thrombus and the layered component.
  - The transition between thrombus and healed tissue had to be continuous, supporting the concept of a “HCP-thrombus continuum”.
4. Localization within culprit lesion segments.
5. No prior stent at the lesion site.
6. High-quality OCT pullback.

The Pilot subgroup served a methodological purpose: the OCT morphological features observed in this subset—particularly the *HCP–healing thrombus continuum*—allowed us to identify and categorize in vivo three distinct *OCT imaging appearances* of HCP, depending on the optical and structural characteristics of the underlying tissue (fibrous, lipid, or calcific). A signal-rich ring/arch was identified when there was high backscattering at the HCP-underlying plaque interface.

- Type I HCP was defined as an abluminal semilunar tissue with moderate backscattering, in comparison to the underlying fibrous plaque;
- Type II HCP was characterized by healing tissue overlying an LRP with or without a signal-rich ring demarcating the two tissues;
- Type III HCP was assigned when the underlying plaque showed calcification at the interface with the healing layer, with or without a signal-rich ring.

Therefore, the Pilot subgroup allowed us to link OCT details to a healing thrombus, in spite of lacking histological data.

Following this, the presence of HCP was determined at the culprit site and non-culprit segments in the entire group of patients, according to the morphological patterns identified in the Pilot group. Culprit HCPs were investigated in terms of plaque length, stenosis severity, underlying plaque morphology, HCP-tissue interface aspect, ACS causative mechanism, or whether the plaque was located in a critical coronary segment (i.e., bifurcation or ostium).

Plaque length was defined as the distance between the proximal and distal ends of the diseased segment. A gap of a minimum of 5 mm was required to consider two lesions as discrete. Cross-sectional plaque frames were analyzed at 1 mm intervals. Corresponding angiographic images using references such as side branch take-off, calcification, or prior stents were useful to properly locate the culprit site.

The reference lumen was identified as the site with the largest lumen area within 10 mm distance to the plaque, but in the same segment. The mean between the proximal and distal references was calculated. Minimal lumen area (MLA) was defined as the smallest area in the lesion segment. Percent area stenosis (AS) was determined using the formula: [(average reference LA—MLA)/average reference LA] × 100 [5]. Following intravascular imaging, coronary lesions were dichotomized as significant or non-significant based on %AS values: >70% or <50%, respectively.

The mechanism of culprit plaque complication was evaluated: PR, PE, or CN. PR was typically defined by the presence of fibrous cap discontinuity with clear cavity formation [4]. PE was generally diagnosed when there was a thrombus or luminal irregularity with an intact fibrous cap [4]. A CN was categorized as eruptive when there was expulsion of small calcific nodules into the lumen and protruding when there was a protruding calcium mass into the lumen in the absence of eruptive nodules [20].

### 2.4. Statistical Analysis

Statistical analysis was performed using MedCalc version 23.1.6 (MedCalc Software Ltd., Ostend, Belgium) from a Microsoft Excel 2021 database. Continuous data were tested for normal distribution using the D’Agostino–Pearson test. Normally distributed variables were expressed as mean ± standard deviation and were compared using the independent sample Student’s *t*-test when there were two groups and ANOVA when there were more than two groups. Non-normally distributed variables were expressed as median (25th to 75th percentiles) and were compared using a Mann–Whitney U test in the case of two groups and a Kruskal–Wallis test when there were more than two groups. Categorical variables were presented as counts (percentages) and were compared using the chi-square test. All tests were two-sided, and a *p*-value < 0.05 was considered statistically significant.

## 3. Results

### 3.1. Pilot Group

A subgroup including 27 patients (34.6%) expressed a continuum between HCP and large fresh thrombus. In 6 patients, the HCP overlayed fibrous tissue and was therefore referred to as a type I, while type II HCP was observed in 17 patients. The remaining four patients had HCP superimposed on calcium deposits, defined as a type III HCP.

Compared to the remaining culprit-HCP cohort based on clinical characteristics, there was a higher incidence of diabetes mellitus (44.4 vs. 19.6%, *p* = 0.02), smoking (66.7 vs. 41.2%, *p* = 0.03), and previous stroke (18.5 vs. 3.9%, *p* = 0.03). On OCT images there was increased lesion severity in patients with an HCP-fresh thrombus continuum (MLA—1.22 vs. 2.17 mm^2^, *p* = 0.01; AS—83 vs. 70%, *p* = 0.0015) (Table 1).

### 3.2. Primary Analysis

Thereafter, on OCT analysis, an HCP pattern was identified at the culprit site in 78 out of 87 (89.7%) ACS patients. The culprit-HCP cohort constituted the main focus of the current study.

There were no differences between patients with or without HCP at the culprit site regarding baseline characteristics. Patients without HCP at the culprit lesion showed instead a significantly higher prevalence of HCP at other segments of the culprit vessel (88.9 vs. 32.1%, *p* = 0.0009).

In the culprit-HCP cohort, mean patient age was 60.2 years, 61.5% were male, and 58 (74.3%) were admitted for NSTE-ACS. Compared to patients with STEMI, those with NSTE-ACS were older (*p* = 0.01), more often hypertensive (*p* = 0.02), more frequently pretreated with Aspirin (*p* = 0.007)/Statin (*p* < 0.001), had more multivessel disease (*p* = 0.03), and showed a greater burden of calcific plaques (*p* = 0.03) and type III HCP (*p* = 0.01). Despite these differences, prior MI and PCI rates were not significantly different.

**Figure 2:**
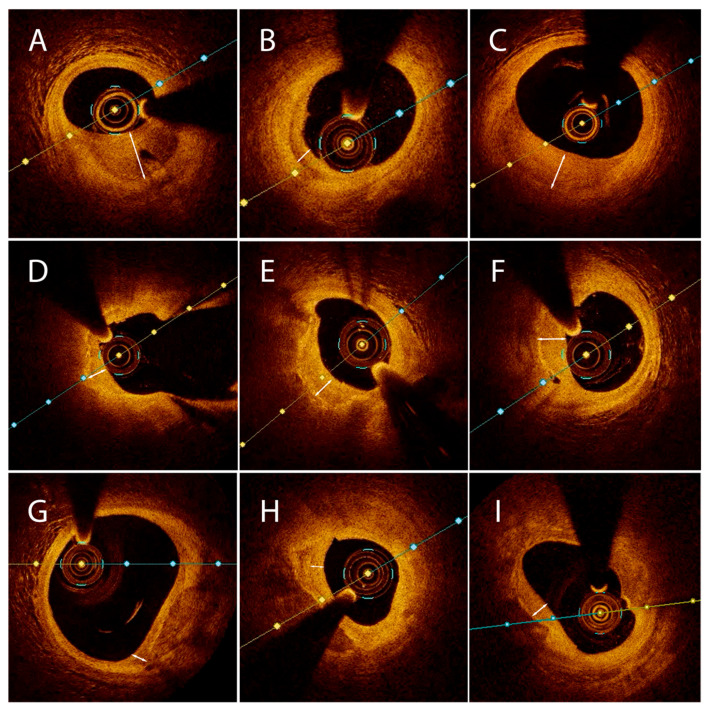
Representative optical coherence tomography images of the three main healed plaque morphologies. (**A**–**C**) Healed plaques overlaying fibrous tissue. (**D**–**F**) Healed plaques overlaying lipid tissue. (**G**–**I**) Healed plaques overlaying calcific tissue. Two-headed arrows indicate the layer of healed lesion. A distinct hyperlucent ring is observed at the healed underlying tissue interface in (**D**–**F**) and (**G**–**I**) but not in (**A**–**C**).

HCPs were categorized into the three types (Figure 2) based on the aforementioned morphological details established in the Pilot group: type I was seen in 24 patients (30.8%), type II in 40 patients (51.3%), and type III in 14 patients (17.9%), respectively.

We found an overall number of 79 non-culprit HCP: 41.8% type I, 39.2% type II, 19% type III (*p* = 0.27 vs. culprit HCP).

#### 3.2.1. Clinical Data

Baseline patient characteristics between the three culprit lesion HCP types are shown in Table 2. There was a higher frequency of NSTE-ACS, which was especially the case for patients with type II and III aspects (80 and 92.9%, *p* = 0.01). Regarding the cardiovascular risk profile, patients in the type III cohort were more frequently overweight (71.4%, *p* = 0.03). No significant differences were found in terms of demographics, history of cardiovascular disease, or medications between the three groups.

#### 3.2.2. Coronary Angiography and OCT Data

When comparing angiographic characteristics (Table 3) between the three culprit site HCP types, type I HCP was more frequently non-significant (45.8%), type II HCP was more commonly significant (60%), while type III HCP presented more frequently as a borderline lesion (42.8%, *p* = 0.05).

**Figure 3:**
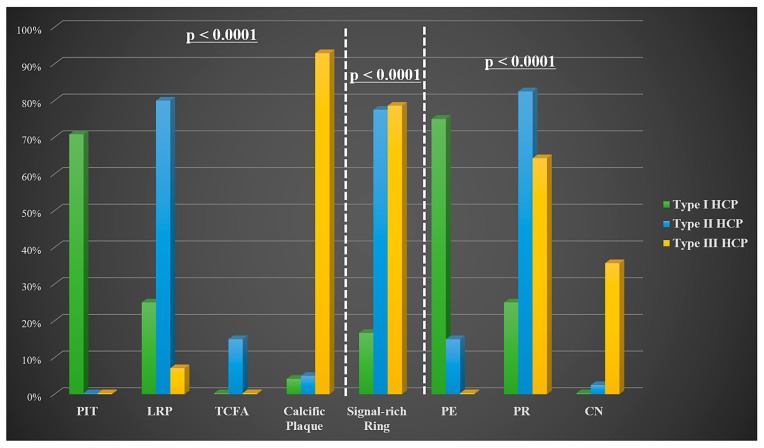
Culprit plaque characteristics between the three different healed plaque morphologies.

OCT analysis (Figure 3) revealed that with respect to the underlying tissue morphology at the culprit site, type I HCP was associated with PIT (70.8%), type II HCP was associated with LRP (80%), while type III HCP was associated with calcific plaque (92.9%, *p* < 0.0001). A signal-rich arch was observed at the HCP-underlying tissue interface in the type II (77.5%) and III (78.6%, *p* < 0.0001) cohorts, while type I rarely expressed this finding. PE was more frequently the precursor lesion to the type I HCP (75%), while PR determined mostly type II HCP (82.5%, *p* < 0.0001), and, in a smaller proportion, type III HCP (64.3%). Type III HCP tended to have longer culprit lesions (*p* = 0.09). Although not statistically significant, type II HCP had lower MLA and higher AS compared to type III and type I HCP (Table 3).

### 3.3. Secondary Analysis

Post-stenting OCT imaging was available in 40 patients from the initial cohort. In addition, a separate subgroup of 29 patients, not included in the primary analysis, underwent OCT examination exclusively after stent implantation. Thus, a total of 69 post-stenting OCT assessments were analyzed to evaluate the presence of ED at stent landing zones (LZ). EDs were identified in 25 cases (36.2% of the stented patients). Among these, 20 cases (80%) had at least one HCP present within the stent LZ. Overall, HCPs were observed in the LZ of 29 patients (42%), of whom 20 developed post-stenting ED (68.9%). There was a significant correlation between ED and HCP presence at LZ (HR 4.14, 95% CI: 1.79–9.55; *p* < 0.001). None of the observed EDs required any additional treatment.

## 4. Discussion

In this in vivo OCT study conducted in a real-world population of patients with ACS, we investigated the prevalence, morphological features, and clinical significance of culprit-HCP. The main findings of the current study are as follows: (1) We provided a detailed OCT characterization of HCP informed by the analysis of fresh intracoronary thrombi at various stages of organization and healing. Based on the morphology of the underlying plaque substrate, HCP could be classified into three distinct imaging types. Notably, type II and type III HCP displayed a characteristic signal-rich interface ring, enhancing their detectability. (2) The detection of HCP in ACS patients was significantly improved using this approach, revealing a high prevalence of HCP at culprit lesion sites. HCPs were present in the majority of these lesions. (3) HCPs located at stent LZs were frequently associated with post-procedural ED, indicating a possible mechanical vulnerability at these sites.

Pivotal studies conducted by Rittersma et al. and later by Kramer et al. [21,22] created a paradigm shift in the understanding of coronary plaque instability and the temporal evolution of thrombosis. In STEMI patients (<6 h after onset of symptoms), coronary thrombus aspiration revealed three distinct stages of maturation: fresh, lytic (1–5 days), and organized (>5 days). Interestingly, thrombus healing was evident in over 50% of patients [21]. Furthermore, in sudden death victims [22], thrombus healing incidence is even higher (69%), indicating that coronary occlusion is not necessarily an abrupt event. Instead, it may often be preceded by a period of subclinical plaque instability.

These studies also highlighted the role of the underlying plaque morphology in influencing thrombus behavior. In the same autopsy study [22], 50% of thrombi associated with PR showed a lack of histological healing (i.e., were fresh), while in contrast, the majority of thrombi associated with PE (>85%) expressed late stages of healing. This may be due to the fact that PE lesions are usually less severe, and so thrombi have more time to evolve and organize.

In our study, in vivo OCT analysis revealed that 34.6% of ACS patients exhibited a combination of HCP and fresh thrombus at the culprit lesion. Building on the analysis of patients with intracoronary thrombi in different phases of organization, we identified three distinct HCP imaging morphologies, classified according to the characteristics of the directly adjacent underlying tissue. The prevalence of each category reflects the mechanism that has led to plaque complications. These patients had a higher incidence of diabetes mellitus and smoking, which are known to be associated with a higher risk of thrombosis [23,24]. In a prothrombotic milieu, local thrombolytic mechanisms will be surpassed, leading to the formation of a mural thrombus. A history of stroke was more common in patients with an HCP-thrombus dyad. This emphasizes the increased vulnerability of this patient subset, with an increased predisposition to multiple vascular complications. There was increased lesion severity in patients with an HCP-thrombus continuum in our study. Similarly to our results, previous work by Kramer et al. [22] found that percent stenosis increased in lesions with fresh thrombi, compared to those with more organized thrombi.

Importantly, the optical properties of the HCP—characterized by collagen-rich tissue with low light attenuation—enabled visualization of the plaque substrate, thereby facilitating the identification of key pathological mechanisms such as PR, PE, or CN. Type I HCP (30.8%), overlaying fibrous tissue, is mainly associated with PIT and results from PE. PE represents the second cause of coronary thrombosis seen in up to 31% of ACS cases [25]. OCT data suggest that PE occurs with a slightly higher frequency on PIT compared to LRP [25,26]. Type II HCP (51.3%), overlaying lipid tissue, is strongly correlated with LRP and arises in the context of PR, the most common precipitating event in ACS (over 43%), as shown by OCT studies [25]. The substrate is always an LRP (with an increased frequency of TCFA), but never a PIT [25,26]. Type III HCP (17.9%), overlaying calcific tissue, is strongly associated with calcific plaque and is determined both by PR and CN. This observed dual nature seems easily understandable since a calcific plaque may contain superficial lipid pools prone to PR as well as superficial calcium that may protrude into the lumen and/or break [27]. The longer plaques observed in this cohort may reflect greater intraplaque heterogeneity.

In this study, patients with a type II culprit HCP presented with a higher degree of stenosis, indicating a greater plaque burden. Traditionally, coronary plaque progression has been considered a gradual process, eventually leading to critical luminal narrowing and ACS [28]. Pathology data by Mann and Davies [2] on PR-induced HCP reported, however, that the majority of plaques with >50% stenoses had an HCP pattern, challenging the concept of gradual progression. More recent serial OCT data also show that in two-thirds of cases, coronary plaques undergo a rapid phasic pattern of growth [29], reinforcing the idea that repetitive episodes of subclinical plaque disruption and healing play a fundamental role in atherosclerosis progression.

HCPs represent the hallmark of previous plaque disruption, followed by silent thrombus healing leading to a layered morphological aspect [3]. The organizing thrombus, rich in platelets and fibrin, is infiltrated by granulation tissue responsible for the production of proteoglycans and type III collagen. As healing progresses, type III collagen is slowly replaced by type I collagen, ultimately leading to full endothelization and new fibrous layer formation [30]. Otsuka et al. [4] proposed that the formation of type I collagen is responsible for the ring of high-backscattering signal visible on OCT. Our research shows that a signal-rich arch was associated with type II and type III HCP, but not with type I HCP, suggesting that this feature is influenced by the underlying plaque morphology. Souteyrand et al. [31] conducted a small serial OCT study investigating plaque healing following an ACS. Patients with an intact fibrous cap (i.e., mainly fibrous plaques) did not demonstrate a hyperlucent arch at any given time throughout the follow-up period. We thereby speculate that for a signal-rich arch to become apparent, an essential requirement is the occurrence of an interface between two tissues with different refractive indices, such as HCP-lipid (type II) and HCP-calcium (type III), respectively.

Previous autopsy studies report that HCP arising in the context of PR has been found in 61–70% of sudden cardiac death subjects [2,3]. More recently, Fracassi et al. [9] found that the in vivo prevalence of HCP at the culprit site was 29% in a cohort of 376 ACS patients. In another OCT study conducted by Wang et al. [10], an HCP phenotype at the culprit lesion was identified in 62% of ACS patients. In the current study, an OCT-identified HCP was found at the culprit lesion in over 89% of ACS cases caused by either PE, PR, or CN. Most of our patients presented with NSTE-ACS, and consistent with existing literature, this group demonstrated a more complex risk profile and a more advanced and extensive atherosclerotic burden. These findings may support the hypothesis that HCP in NSTE-ACS patients represent a more mature, possibly subacute phase of plaque destabilization, contrasting with the more abrupt nature of STEMI, potentially allowing time for healing and organization of the disrupted plaques. This may in part explain the high prevalence of HCP observed in our study. Several additional factors may account for this finding. First, differences in methodology across prior research—including only ACS due to PR [2,3] or excluding calcium-related acute events—may have led to underreporting [9,10]. Second, a better understanding of the mechanisms of coronary plaque progression and increasing awareness of this particular entity have led to superior physician HCP detection capabilities. Third, the detailed OCT characterization of HCP derived from our group of patients with fresh thrombus enabled the identification of a broad spectrum of HCP, many of which might have otherwise gone unrecognized. It should be noted, however, that the present study emphasizes the existence of different imaging aspects of the same pathological entity, HCP, rather than describing distinct pathological processes.

OCT-detected stent EDs are associated with impaired outcomes at long-term follow-up [32]. In our study, we observed not only a high burden of overall HCP but also a high rate of stent ED when HCP was detected at the LZ, thus suggesting HCP may play a role in procedural-related outcomes. Enhanced HCP detection through our newly proposed classification system may therefore aid in stratifying patients at higher risk for stent edge-related complications during percutaneous coronary interventions while also contributing to improved stent and ultimately clinical results through better LZ selection or by choosing longer stent lengths. We hypothesize that a potential mechanism for ED related to HCP may stem from intrinsic structural features of these plaques, specifically, an increased propensity for cleavage plane formation at the interface between HCP and the underlying plaque substrate. This interface may render the arterial wall more susceptible to mechanical disruption during stent deployment. However, in many of our cases, the dissection plane extended along the media layer (beneath the whole plaque) despite the entry point being located at the margins of the HCP. Thus, while the interface between HCP–substrate represents an intuitive site of mechanical failure, the presence of HCP may primarily serve as a marker of the structural vulnerability of the underlying culprit plaque, rather than acting as a direct cause of dissection.

**Figure 4:**
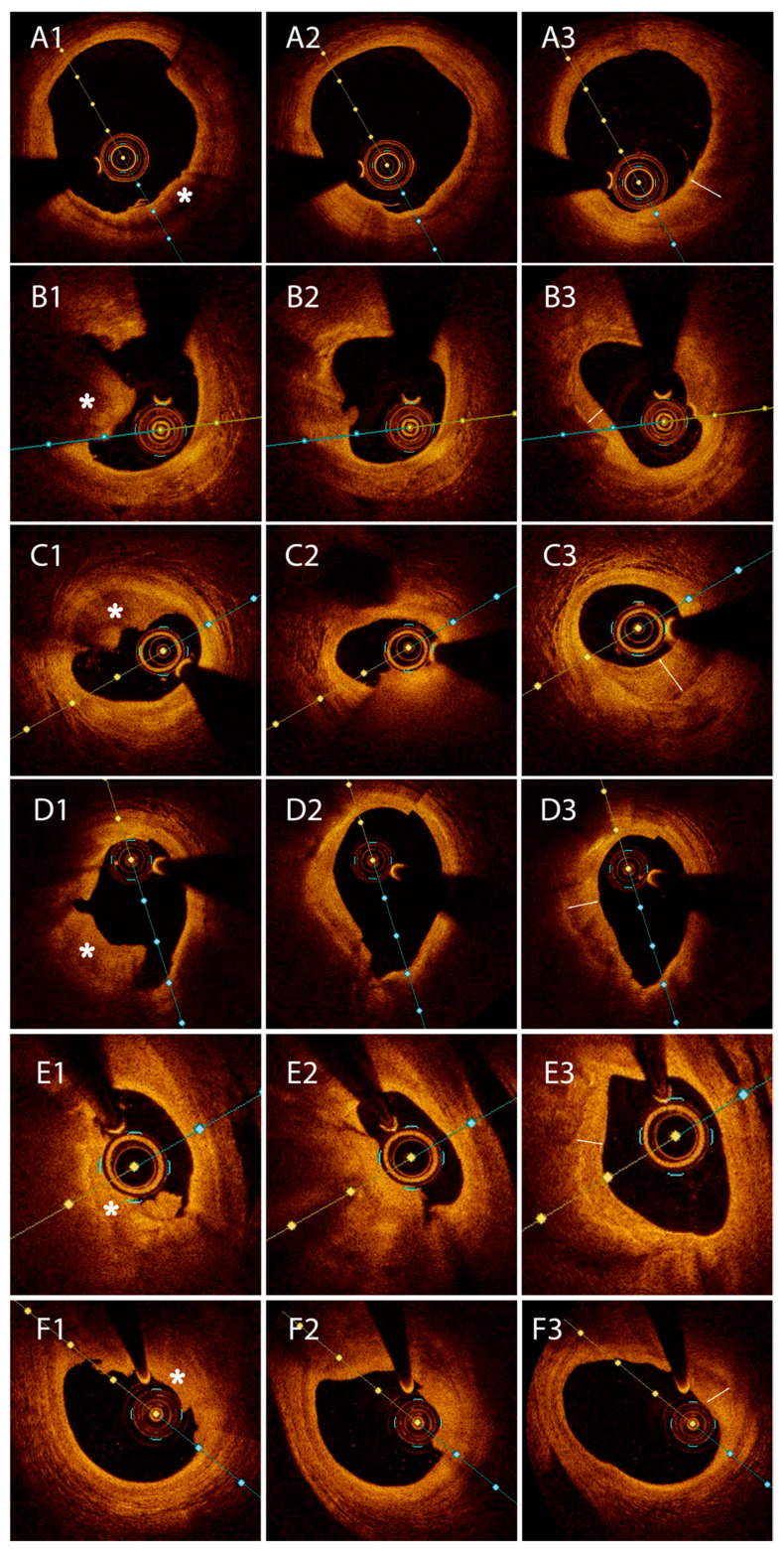
Optical coherence tomography images from six different patients (**A1**–**F3**) exhibiting a continuum of healed plaque-fresh thrombus at the culprit site at the same point in time. Two-headed arrows indicate a healed plaque component. Asterisks indicate thrombus components.

In the present study, although not reaching statistical significance, there is increased patient age between type I vs. type II vs. type III culprit HCP, which also applies to plaque length. Additionally, lesion severity tends to increase when comparing type II vs. type III vs. type I HCP. These findings emphasize the ubiquitous nature of HCP, impacting patients in different age groups and associating with lesions ranging from less complex and fibrotic to more complex and mature that are more calcific in nature. Moreover, a higher prevalence of non-culprit HCP was observed in patients without HCP at the culprit site compared to the culprit-HCP cohort, suggesting a chronic and diffuse pattern of coronary vulnerability and supporting the notion that these groups likely do not represent fundamentally different populations. All of the aforementioned aspects, together with the increased HCP prevalence seen in our study, suggest that all non-occlusive thrombi most probably suffer a process of healing, which in turn is a fundamental mechanism involved in coronary plaque progression. Another argument favoring this hypothesis is provided by our Pilot group, in which we identified fresh thrombus, and within the thrombus (typically at the terminal segments), an HCP component (crescent shape with a smooth luminal edge) (Figure 4). In one of our patients, OCT imaging performed 8 days after an acute STEMI (Figure S1) revealed the presence of thrombus with signs of healing, which is in concordance with pathological studies that observed that healing starts > 5–7 days after thrombus formation [21,22].

Several limitations of the current study should be considered. First, this is a retrospective single-center study with a small sample size; consequently, the results should be interpreted with caution and are hypothesis-generating but pave the road for future prospective studies; the possible link between HCP detection and procedural complications, such as stent ED needs, biomechanical and histopathological validation. Second, clinical information regarding physical activity and non-modifiable or non-traditional cardiovascular risk factors was not available. Third, cases with poor image quality or where only post-interventional imaging was available were excluded, which created a selection bias. Fourth, another selection bias resides in the interventional cardiologists’ decision to perform OCT—OCT was more likely to be performed on the left anterior descending artery, as this vessel has prognostic implications. OCT was less likely to be performed in acute STEMI patients, as a large thrombotic mass would discourage intravascular imaging use; some of them were in fact patients with transient ST-segment elevation and ambiguous culprit lesions at the time of coronary angiography, hence the larger incidence of STEMI in the type I-HCP cohort. Additionally, OCT was more likely to be performed in case of borderline severity lesions, for example, on the non-culprit vessel. Fifth, OCT differential diagnosis may sometimes be cumbersome, for instance, when discriminating between CN and red thrombus, due to high lesion attenuation. Similarly, HCP overlaying superficial calcified plates and generating a hyperlucent ring were occasionally difficult to differentiate from the macrophage infiltration seen in TCFA. In both cases, adjacent frames were reviewed, and the diagnosis of CN or superficial calcified plates was confirmed when detecting additional dense calcifications. Image analysis was performed without the use of dedicated CoreLab software. Sixth, a matter of debate remains the temporal relation between an acute event and the characteristic HCP phenotype, as events occurring in the distant past may no longer be recognizable. Finally, clinical and imaging follow-up was beyond the scope of the current investigation.

## 5. Conclusions

The detailed OCT-based analysis of intracoronary organizing thrombi enabled in vivo classification of culprit HCP into distinct imaging subtypes, contributing to a high detection rate. HCP can create mechanical vulnerability if located at the stent LZ and may predict post-procedural ED. Our refined HCP detection techniques may help optimize stent-related outcomes of OCT-guided interventions by enabling more precise LZ selection in areas devoid of HCP or by choosing longer stents.

## Figures and Tables

**Table 1:** Baseline, angiographic, and OCT characteristics between patients with culprit HCP-thrombus continuum vs. those with HCP-only.

Variable	Culprit HCP-Thrombus (n = 27)	Culprit HCP-Only (n = 51)	*p*-Value
Age, years	59.4 ± 11.9	60.6 ± 10.1	0.641
Male gender	18 (66.7)	30 (58.8)	0.5
**Diagnosis**			0.356
NSTE-ACS	18 (66.7)	39 (76.5)	
STEMI	9 (33.3)	12 (23.5)	
**Risk factors**			
Hypertension	22 (81.5)	42 (82.4)	0.924
Diabetes mellitus	12 (44.4)	10 (19.6)	0.021
Previous hyperCHOL	18 (66.6)	39 (76.4)	0.353
Smoking habit	18 (66.7)	21 (41.2)	0.033
Overweight	9 (33.3)	25 (49.0)	0.186
Severe * CKD	3 (11.1)	2 (3.9)	0.217
**Clinical history**			
Previous MI	8 (29.6)	18 (35.3)	0.615
Previous PCI	6 (22.2)	11 (21.6)	0.947
Previous stroke	5 (18.5)	2 (3.9)	0.033
Previous CHF	2 (7.4)	8 (15.7)	0.301
Atrial fibrillation	2 (7.4)	6 (11.8)	0.548
**Laboratory data**			
LDL-C, mg/dL	109.1 ± 33.7	121.7 ± 40.2	0.171
HDL-C, mg/dL	40 (34.5–47.2)	42 (32.5–48.7)	0.841
Triglycerides, mg/dL	125 (89.2–203.7)	140 (91.2–210)	0.559
Creatinine, mg/dL	1 (0.7–1.3)	0.9 (0.8–1.1)	0.776
Peak CK-MB, U/L	23 (12.7–70.2)	25.8 (15–62)	0.92
Peak hs-cTnI, ng/L	72 (38–4665.7	55 (30.5–3580)	0.394
NT-proBNP, pg/mL	233 (51.5–942)	133 (44.2–410.7)	0.341
Leucocytes, 10^9^/L	9.3 (6.8–10.8)	8.2 (6.3–9.2)	0.115
Ne-Ly ratio	2.9 (2.3–4.3)	3.1 (2.3–4.2)	0.979
CRP, mg/dL	0.4 (0.3–1.2)	0.4 (0.1–1.9)	0.389
Hemoglobin, g/dL	13.9 (12.3–14.8)	14.1 (12.9–14.6)	0.360
**Echocardiography data**			
LVEF, %	50 (40.7–50)	50 (48–55)	0.382
LVEDD, mm	48 (46.25–51.7)	48 (44.2–51)	0.720
**Previous medication**			
Aspirin	10 (37)	17 (33.3)	0.745
Statin	20 (74.1)	37 (72.5)	0.885
ACEI/ARB	20 (74.1)	36 (70.6)	0.746
**Angiographic data**			
*Culprit vessel*			0.225
LM	2 (7.4)	1 (2.0)	
LAD	23 (85.2)	46 (90.2)	
LCX	0 (0)	3 (5.9)	
RCA	2 (7.4)	1 (2)	
Multivessel disease *	12 (44.4)	19 (37.3)	0.539
*Lesion severity*			0.05
Non-significant	3 (11.1)	17 (33.3)	
Borderline	4 (14.8)	12 (23.5)	
Significant	17 (63)	20 (39.2)	
Occlusive	3 (11.1)	2 (3.9)	
**OCT data**			
*Initial lesion*			0.711
Plaque erosion	8 (29.6)	16 (31.4)	
Plaque rupture	16 (59.3)	32 (62.7)	
CN (protrusive/eruptive)	3 (11.1)	3 (5.9)	
*Underlying plaque morphology*			0.992
PIT	6 (22.2)	11 (21.6)	
LRP	13 (48.1)	26 (51)	
TCFA	2 (7.4)	4 (7.8)	
Calcific plaque	6 (22.2)	10 (19.6)	
Signal-rich arch at tissue interface	14 (51.9)	32 (62.7)	0.355
Ostial HCP involvement	7 (25.9)	8 (15.7)	0.278
Bifurcation HCP involvement	19 (70.4)	32 (62.7)	0.503
RLA, mm^2^	7.0 (5.4–8.4)	7.5 (6.12–8.7)	0.756
MLA, mm^2^	1.2 (0.9–2.1)	2.1 (1.3–3.9)	0.013
Area stenosis, %	83 (71.7–83.7)	70 (53.2–79.7)	0.001
Lesion length, mm	26.0 ± 8.8	22.3 ± 10.8	0.128
Non-culprit HCP on culprit vessel	7 (25.9)	18 (35.3)	0.402

Data are presented as n (%), mean ± SD, or median (25th and 75th percentile). ACEI: angiotensin-converting enzyme inhibitor. ARB: angiotensin receptor blocker. CHF: chronic heart failure. CKD: chronic kidney disease. CK-MB: creatine-kinase MB isoform. CN: calcified nodule. CRP: C-reactive protein. HCP: healed coronary plaque. HDL-C: high-density lipoprotein cholesterol. hs-cTnI: high-sensitivity Troponin I. hyperCHOL: hypercholesterolemia. LAD: left anterior descending artery. LCX: left circumflex artery. LDL-C: low-density lipoprotein cholesterol (range 70–130 mg/dL). LM: left main. LRP: lipid-rich plaque. LVEDD: left ventricular end-diastolic diameter. LVEF: LV ejection fraction (normal 50–70%). MI: myocardial infarction. MLA: minimal lumen area. Ne-Ly: neutrophil-lymphocyte. NSTE-ACS: non-ST-segment elevation acute coronary syndromes. NT-proBNP: N-terminal pro b-type natriuretic peptide. PCI: percutaneous coronary intervention. PIT: pathological intimal thickening. STEMI: ST-segment elevation MI. RCA: right coronary artery. RLA: reference lumen area. TCFA: thin-cap fibroatheroma. * Severe CKD is defined as a glomerular filtration rate < 30 mL/min/1.73 m^2^.

**Table 2:** Baseline patient characteristics between the three culprit site HCP morphologies.

Variable	HCP Type I (n = 24)	HCP Type II (n = 40)	HCP Type III (n = 14)	*p*-Value
Age, years	57.3 ± 11.3	60.8 ± 10.3	63.5 ± 10.5	0.204
Male gender	14 (58.3)	24 (60)	10 (71.4)	0.696
**Diagnosis**				0.01
NSTE-ACS	13 (54.2)	32 (80)	13 (92.9)	
STEMI	11 (45.8)	8 (20)	1 (7.1)	
**Risk factors**				
Hypertension	20 (83.3)	32 (80)	12 (85.7)	0.874
Diabetes mellitus	9 (37.5)	10 (25)	3 (21.4)	0.462
Previous hyperCHOL	15 (62.5)	28 (70)	10 (71.4)	0.786
Smoking habit	11 (45.8)	21 (52.5)	6 (42.9)	0.778
Overweight	7 (29.2)	17 (42.5)	10 (71.4)	0.039
Severe * CKD	1 (4.3)	3 (7.5)	1 (7.1)	0.882
**Clinical history**				
Previous MI	9 (37.5)	11 (27.5)	5 (35.7)	0.672
Previous PCI	7 (29.2)	8 (20)	2 (14.3)	0.521
Previous stroke	1 (4.2)	5 (12.5)	1 (7.1)	0.510
Previous CHF	3 (12.5)	4 (10)	3 (21.4)	0.544
Atrial fibrillation	2 (8.3)	3 (7.5)	3 (21.4)	0.312
**Laboratory data**				
LDL-C, mg/dL	115.0 ± 43.5	121.2 ± 34.9	110.4 ± 40.1	0.629
HDL-C, mg/dL	41 (29.5–46.5)	39.5 (34.5–46)	46 (33–55)	0.516
Triglycerides, mg/dL	128 (85–217)	131.5 (90.5–187.5)	149.5 (125–293)	0.302
Creatinine, mg/dL	0.8 (0.8–1.1)	1.0 (0.8–1.1)	0.9 (0.8–1.0)	0.565
Peak CK-MB, U/L	36.5 (17.3–67)	23 (11.5–55.5)	18 (15–62)	0.276
Peak hs-cTnI, ng/L	1866.5 (37–4088.5)	44 (927–3688.5)	42.5 (33–3570)	0.353
NT-proBNP, pg/mL	271.5 (50–886.5)	127.5 (43–366)	151.5 (78–560)	0.762
Leucocytes, 10^9^/L	8.52 (6.3–11.0)	8.3 (6.8–10.4)	6.6 (5.7–8.4)	0.086
Ne-Ly ratio	3.07 (1.7–4.4)	2.9 (2.3–4.4)	3.05 (2.3–3.8)	0.716
CRP, mg/dL	0.47 (0.1–3.8)	0.42 (0.2–1.1)	0.4 (0.1–2.2)	0.901
Hemoglobin, g/dL	13.8 (12.7–14.5)	14.1 (12.9–14.6)	14.05 (13.3–15)	0.639
**Echocardiography data**				
LVEF, %	50 (47–50)	50 (46–55)	50 (50–55)	0.588
LVEDD, mm	48 (44.5–54)	48 (44–51)	50 (48–52)	0.057
**Previous medication**				
Aspirin	9 (37.5)	13 (32.5)	6 (42.9)	0.770
Statin	15 (62.5)	31 (77.5)	11 (78.6)	0.372
ACEI/ARB	15 (62.5)	29 (72.5)	12 (85.7)	0.305

Data are presented as n (%), mean ± SD, or median (25th and 75th percentile). ACEI: angiotensin-converting enzyme inhibitor. ARB: angiotensin receptor blocker. CHF: chronic heart failure. CKD: chronic kidney disease. CK-MB: creatine-kinase MB isoform. CRP: C-reactive protein. HCP: healed coronary plaque. HDL-C: high-density lipoprotein cholesterol. hs-cTnI: high-sensitivity Troponin I. hyperCHOL: hypercholesterolemia. LDL-C: low-density lipoprotein cholesterol (range 70–130 mg/dL). LVEDD: left ventricular end-diastolic diameter. LVEF: LV ejection fraction (normal 50–70%). MI: myocardial infarction. Ne-Ly: neutrophil-lymphocyte. NSTE-ACS: non-ST-segment elevation acute coronary syndromes. NT-proBNP: N-terminal pro b-type natriuretic peptide. PCI: percutaneous coronary intervention. STEMI: ST-segment elevation MI. * Severe CKD is defined as a glomerular filtration rate < 30 mL/min/1.73 m^2^.

**Table 3:** Angiographic and OCT characteristics between the three culprit site HCP morphologies.

Variable	HCP Type I (n = 24)	HCP Type II (n = 40)	HCP Type III (n = 14)	*p*-Value
**Angiographic data**				
*Culprit vessel*				0.255
LM	0 (0)	1 (2.5)	2 (14.3)	
LAD	21 (87.5)	36 (90)	12 (85.7)	
LCX	1 (4.2)	2 (5)	0 (0)	
RCA	2 (8.3)	1 (2.5)	0 (0)	
Multivessel disease *	6 (25)	17 (42.5)	8 (57)	0.130
*Lesion severity*				**0.05**
Non-significant	11 (45.8)	8 (20)	1 (7.1)	
Borderline	3 (12.5)	7 (17.5)	6 (42.9)	
Significant	10 (41.7)	24 (60)	7 (50)	
Occlusive	0 (0)	1 (2.5)	0 (0)	
**OCT data**				
Ostial HCP involvement	3 (12.5)	9 (22.5)	3 (21.4)	0.6
Bifurcation HCP involvement	14 (58.3)	27 (67.5)	10 (71.4)	0.659
RLA, mm^2^	8.2 (6.5–9)	7.0 (6.1–8.1)	7.9 (5.4–9.4)	0.454
MLA, mm^2^	2.8 (1.0–4.3)	1.4 (1.1–2.6)	1.8 (1.1–3.0)	0.586
Area stenosis, %	67 (51–82.5)	76.5 (64.5–83)	71.5 (67–83)	0.404
Lesion length, mm	21.5 ± 9.5	23.1 ± 9.3	28.7 ± 13.0	0.098
Non-culprit HCP on culprit vessel	8 (33.3)	16 (40)	1 (7.1)	0.075

Data are presented as n (%), mean ± SD, or median (25th and 75th percentile). HCP: healed coronary plaque. LAD: left anterior descending artery. LCX: left circumflex artery. LM: left main. MLA: minimal lumen area. RCA: right coronary artery. RLA: reference lumen area. * defined as the presence of a significant stenosis in any of the non-culprit vessels or involvement of the left main.

## Data Availability

The data presented in this study are available on request from the corresponding author. The data are not publicly available because they are the property of Cluj County Emergency Hospital, Cluj-Napoca, Romania.

## Supplementary Materials

The following supporting information can be downloaded at: https://www.mdpi.com/article/10.3390/medicina61081440/s1, Figure S1: Patient with acute ST-segment elevation myocardial infarction. (A) Index coronary angiogram. (B) Repeat coronary angiogram after 8 days of conservative treatment. (a–c) Corresponding OCT images showing fresh thrombus distally (c) which becomes progressively more organized in the proximal segments (b to a). Two-headed arrow indicate the layer of healed thrombus

## Abbreviations

ACS	Acute coronary syndrome
AS	Percent area stenosis
CAD	Coronary artery disease
CN	Calcified nodule
ED	Edge dissections
HCP	Healed coronary plaques
LRP	Lipid-rich plaque
LZ	Landing zone
MLA	Minimal lumen area
NSTE-ACS	Non-ST-segment elevation acute coronary syndromes
OCT	Optical coherence tomography
PE	Plaque erosion
PIT	Pathological intimal thickening
PR	Plaque rupture
STEMI	ST-segment elevation myocardial infarction
TCFA	Thin-cap fibroatheroma
