# Supplementary material for: OCT-Based Morphological Classification of Healed Coronary Plaques: Insights from Imaging of Fresh Thrombi at Different Stages of Healing and Implications for Post-Stenting Edge Dissections
Source: Medicina (Kaunas). 2025 Aug 10;61(8):1440. doi: 10.3390/medicina61081440 (PMC12387862; doi:10.3390/medicina61081440)
Supplement: Supplementary file 1 [file medicina-61-01440-s001.zip › medicina-3757645-supplementary.pdf]

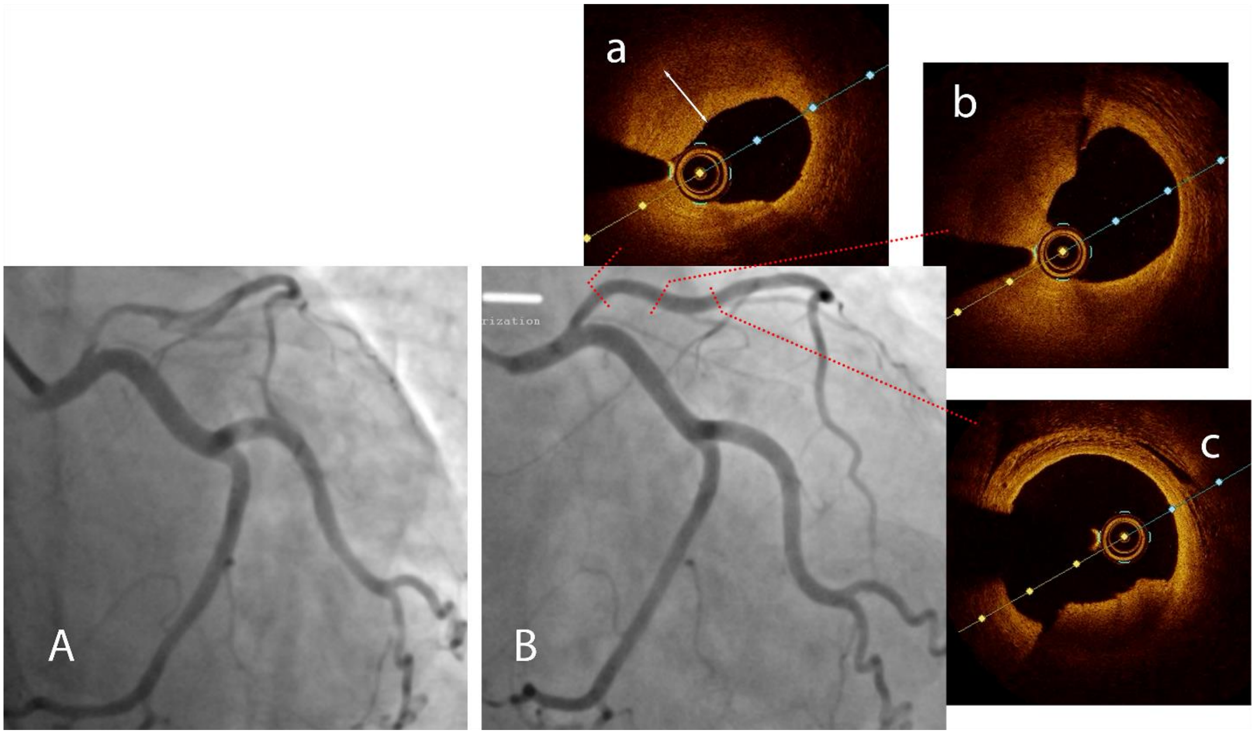

Supplemental Material Figure 1. Patient with acute ST-segment elevation myocardial infarction. (A) Index coronary angiogram. (B) Repeat coronary angiogram after 8 days of conservative treatment. (a-c) Corresponding OCT images showing fresh thrombus distally (c) which becomes progressively more organized in the proximal segments (b to a). Two-headed arrow indicate the layer of healed thrombus
